# Health and the Rural Districts

**Published:** 1920-02-14

**Authors:** 


### HEALTH AND THE RURAL DISTRICTS.

So many of the more widely-published health records come from the larger towns of the country, and so natural is the tendency for the lay press to give space to them according to the size of the community rather than the importance of the particular medical aspect concerned, that the public may often tend to forget how vital to the country are the urgent problems of rural hygiene. It is true that no sharp line can be strictly drawn between rural and urban considerations, for the principles in both are practically identical, and enlightenment in the cities will rapidly spread to the countryside. Nevertheless, whatever affects directly and importantly the residents of our farms and villages is vital to the strength of the nation as a whole, and therefore it may be claimed that the urgency of improvement in rural hygiene is, if not greater, at least as great as that in the case of the town-bred masses.

It is needless to recount the million channels of direct and indirect contact which exist. The vast bulk of milk and fresh food supplies, the actual human influx, the many reservoirs, carefully supervised though they are, which draw their water from the springs and surface of extensive rural territories, and in fact any combination of the three media—persons, food and water—may carry infection from the insanitary rural premises to the city. Daily, hourly in fact, are such infections arriving, and the circumstance that the result is not more alarming is mainly due to the commendable activity of our urban health authorities. The country district has the advantage of a sparser population as an important factor in preventing local spread of communicable disease. On the other hand, the urban district can contrast with this an economic advantage in carrying out mass sanitary measures. But these very measures, upon which the country is at present concentrating so much public approval, are expensive of both money and effort—more costly indeed than the average casual reader will believe. Mass organisation will always be essential to the protection of our cities, but the strain upon its efficiency may be, in some measure at least, relieved by attention to the origin of disease invasion. Yet, in passing through the average small town or rural district, a casual glance from a train window will reveal more often than not insanitary conditions which should be shocking to the average person in these days of so-called enlightenment.

Certainly public notice might be more insistently drawn to the need for improved hygiene throughout the length and breadth of the country, and not in our crowded cities alone. Diseases there are which, caused by infections spread from person to person, are, notwithstanding the more dilute population, much more proportionately prevalent in the apparently healthful environment of our countryside, and we would urge that greater prominence be given to such facts. Many are the popular beliefs founded upon no scientific proof whatever, and in no science is this more apparent than in personal hygiene. In our new section on the Public Well-being will be found an invaluable selection of these points as they occur week by week.

